# Effect of Single-Walled Carbon Nanotubes on Strength Properties of Cement Composites

**DOI:** 10.3390/ma13061305

**Published:** 2020-03-13

**Authors:** Jiayuan Kang, Salam Al-Sabah, Roger Théo

**Affiliations:** School of Civil, Structural & Environmental Engineering, University College Dublin, D04 V1W8 Dublin 04, Ireland; jiayuan.kang@ucdconnect.ie (J.K.); theo.roger@ucdconnect.ie (R.T.)

**Keywords:** single wall carbon nanotubes, surfactant, TritonX-100, mass of specimens, compressive strength, flexural Strength, bulk density

## Abstract

This study aimed to investigate the effects of single-walled carbon nanotubes (SWCNTs) on strength the properties of cement composites when surfactant (SAA) was applied as the dispersion method. TritonX-100 (TX10) was used as the SAA to pretreat SWCNTs, which has been proved to perform well in dispersing the agglomerates of SWCNTs. In this study, four different concentration of SWCNTs, namely 0.00 wt%, 0.02 wt%, 0.04 wt%, and 0.06 wt% by the mass of cement, were used to prepare cement composite specimens. The compressive strength and flexural strength of specimens were tested and recorded. The results show that the compressive and flexural strengths of cement composites decreased with the increase in the concentration of SWCNTs without the addition of TX10. However, when SWCNT suspensions were pretreated with TX10, the strength variation pattern changed; the compressive and flexural strengths of cement composites increased as a function of the concentration of SWCNTs, although there were reductions compared to non-TX10-treated specimens at all concentrations of SWCNTs. Furthermore, the relationship between the strength of cement composites and bulk density of specimens was considered.

## 1. Introduction

Concrete is known to have high compression strength but weak tensile capacity. Many scholars use steel fiber, polyvinyl alcohol (PVA) fiber, polypropylene (PP) fiber, carbon fiber, etc. as micro-reinforcement paste in concrete to prepare composite materials with better bearing capacity [1,2,3,4,5,6,7,8]. The results show that the mix of fiber can effectively enhance the tensile strength and toughness. The problem is that the strength of toughening fibers used in concrete are relatively low, and may cause larger porosity, imposing a negative effect on the mechanical properties of concrete. High strength fibers that are smaller than microfibers for applications of high-performance fiber reinforced concrete composite still has potential. Thus, the feasibility of applying carbon nanotubes (CNTs), as potential nanofibers, into cementitious composites has been widely studied due to CNTs’ unique properties. 

CNTs are known to have spectacular properties, such as high specific conductivity, high Young’s modulus, high yield strength, good thermal properties, etc. [9,10,11,12,13,14,15,16,17,18,19,20,21]. CNTs’ thermal conductivity is at least twice that of diamond [22], and its electricity conductivity is 1000 folds that of copper [12]. Specifically for mechanical properties, CNTs have an average Young’s modulus around 1 TPa, which is approximately five times greater than steel, and the tensile strength is up to 63 GPa which is approximately 50 times greater than steel [12,13,14,15,16,17,19]. CNTs have great potential for reinforcing cementitious composites.

However, when comparing CNTs to traditional microfibers, it is evident that using CNTs in a cement matrix is challenging. One of the main obstacles is the method to disperse CNTs homogeneously in the cement matrix due to their strong van der Waals force (VDW), which originates from their polarizable extended π-electron systems [20], causing CNTs to have a tendency to agglomerate [23,24,25]. This behavior can cause negative effects to the mechanical properties of the cement composites [25]. A homogeneous morphology possesses an improved mechanical resistance, as mentioned by Gavallaro et al. [26] and Lisuzzo et al. [27]. According to Cota et al. [28], the mixing process using a Hobart mixer, commonly used to prepare mortar paste, cannot ensure proper dispersion of CNTs within the cementitious matrix, resulting in large CNT clusters within the hydrated paste. Since the CNTs are not well dispersed in the cement matrix, their spectacular physical properties cannot be fully revealed. The agglomerates and bundles could lead to a decrease in mechanical performance of cementitious materials [25]. Therefore, finding dispersion methods is an important aspect for further study.

Dispersion performance is a key for improving the interface bonding force among CNTs and cement matrix. In previous studies [24,29,30,31,32,33,34], it was found that surfactant (SAA), ultrasonic dispersion, chemical covalent modification, and electricity field induced method are the main methods of dispersing CNT, among which SAA and sonication are the most widely used methods. SAA, as the most widely used method, has many options, such as Sodium dodecyl benzene sulfonate (SDBS), sodium deoxycholate (NaDC), polyethylene glycol octylphenol ether (TX10), Gum Arabic (GA), cetyl trimethyl ammonium bromide (CTAB), and dodecyltrimethyl-ammonium bromide (DTAB), which can make carbon nanotubes disperse and stay stable for a long time.

Many scholars have investigated the possibility and feasibility of introducing CNTs into the field of civil engineering, and have found positive effects of CNTs on cementitious composites. Wang et al. [35] found that the addition of multi-walled carbon nanotubes (MWCNTs), with surfactant GA and sonication treatment, improved the mechanical properties of cement composites. The flexural toughness and fracture energy of cement-based composites were increased with 0.08 wt% addition of MWCNTs. Sindu et al. [36] also found that the combination of GA and sonication can improve the mechanical properties of cement composites. Wang et al. [35] found that, when the concentration of MWCNTs was increased to a certain amount, a negative effect would occur. Wu et al. [37] found that the addition of MWCNTs could significantly relieve the mechanical properties of cement-based composite materials. When the amount of MWCNTs reached its optimal value, i.e., 0.1%, the improvement of the mechanical properties of cement-based composite materials was the best. Fatemi and Foroutan [38] concluded that TX10 was the best at dispersing MWCNTs. Luo et al. [39] found that, by using SDBS and TX10 with sonication, the MWCNT–cement composites saw 29.10% and 20.8% increase in flexural and compressive strength, respectively. Mohamed et al. [40] found that, when sonication was applied to CNT/SAA solution to disperse MWCNTs, higher strength of specimens was obtained. Ana et al. [41] found that the addition of MWCNTs treated with superplasticizer (SP) and sonication improves the compressive strength of cement composites. Similarly, Li et al. [42] concluded that, by dispersing SWCNTs using sonication and SP, a 26.3% increase in strength of cement composites was obtained. Parveen et al. [43] concluded that the compressive strength and flexural strength of mortars saw 19% and 7%, respectively, increase when 1% SWCNTs were dispersed by sonication and Pluronic F-127, a novel dispersing agent. Based on the research presented above, it can be concluded that, when SAA and sonication are combined, a positive effect on the mechanical properties on cement composites can be obtained within a certain range of concentrations of CNTs. 

However, some studies show a slight increase or even a reduction in mechanical properties of CNT–cement composites when SAA and sonication are used. Camacho et al. [44] concluded that the addition of CNT to Portland cement mortars shows little effect on bending strength and apparent density of mortars. Collins et al. [33] found the compressive strength was decreased to four times less compared to a plain cement composite. Similarly, Shao et al. [45] concluded that, when surfactants, namely SDS (Sodium dodecyl sulfate), TX10, and Polyethylene glycol sorbitan monolaurate (Tween-20), and sonication were combined to disperse CNTs in cement matrix, the compressive strength decreased compared to plain cement composites. Sobolkina et al. [46] also found that the use of SDS led to a drop in the strength of cement composites due to the formation of foam. Kim and Chung [47] found that less yield stress was obtained when SWCNTs were treated by applying sonication and SDS or Sodium deoxycholate (DOC). These results conflict with the conclusions obtained in the above studies. 

It is worth mentioning the research of Bharj et al. [48], who found that, by using sonication only (without SAA) to disperse CNTs, it was possible to improve the mechanical properties of cement composite paste. Therefore, sonication probably always has a positive effect on strength for cement composites by dispersing CNTs in matrix, but it should be noted that ultrasonication can result in fragmentation of CNTs and poor stability of dispersion [49]. Therefore, SAA plays an important role in producing a stable CNT dispersion. Different types of SAA can produce either positive or negative effects on cement composites, regardless of their CNT dispersion performance. It has also been found that adding some types of surfactants into cement matrix can cause formation of foam or prevent hydration reaction among cement [45,46,50]. However, few studies have mentioned the formation of foam in sample preparation and the change of rheological behavior of slurry. According to Parveen et al. [43], the surfactant Pluronic F-127 works similarly to polycarboxylate-based superplasticizers, which can disperse cement particles and modify the fluidity of mortar [51]. It is known that plasticizers can keep the slump and cement dosage unchanged while significantly reducing water consumption of mixing additives. However, few studies have considered this and changed the water/cement ratio to keep the slump result constant. Moreover, there are few studies on the effect of using surfactant only to modify the cement composites.

In addition, most studies use MWCNTs as reinforcement materials in cement-based composites [52]. As mentioned above, CNTs have spectacular properties. However, SWCNTs and MWCNTs have many different properties such as Young’s modulus [53,54], electrical conductivity, thermoelectric properties, and optical properties [55]. Table 1 shows the differences between SWCNTs and MWCNTs [53,54].

Table 1 shows that the mechanical properties of MWCNTs are generally better than SWCNTs. This is why most studies use MWCNTs to reinforce cementitious composites, as well as their relative lower cost. Most literature reports are related to mechanical properties. However, in recent years, the needs of different functions of cementitious composites are proposed. Heeyoung et al. [56] investigated the heating characteristics of CNT–cement mortars by using SWCNTs and MWCNTs. They concluded that cement mortars mixed with SWCNTs are more effective for modifying the heating characteristics compared to MWCNTs mixed mortars. However, Luigi et al. [57] found that MWCNT–cement composites showed better electrical properties, which can be applied for future work of monitoring the stress level of concrete element. Heeyooung et al. [58] found that adding CNTs to cement-based materials enhances their electrical and thermal characteristics, and they found that adding SWCNTs to concrete grout is more suitable than MWCNT for identifying voids in the duct through electrical resistance analysis. Jin et al. [59] concluded that extremely small amounts of SWCNTs can be used as optical strain sensors if the SWCNTs can be homogeneously dispersed in cement composites. These studies aimed to fully use the better electrical properties and thermal properties of SWCNTs; however, the mechanical properties of CNTs added to cement-based composites were not considered. For future application, on the premise of meeting the above cementitious composite functions, it is worth studying their mechanical properties. 

This study investigated the effect of different concentrations of SWCNTs on the strength properties of cement composites when SAA dispersion method was used. TX10 was used as SAA because it has shown a higheer dispersing power than SDBS, CTAB, and DTAB for SWCNT suspensions [49]. In addition, the effect of TX10 on strength properties of cement composites was investigated and a comparison of strength of adding SWCNTs and MWCNTs to cement composites after 28 days of curing was also conducted.

## 2. Materials and Methods

### 2.1. Materials

#### 2.1.1. Carbon Nanotubes

**Figure 1:**
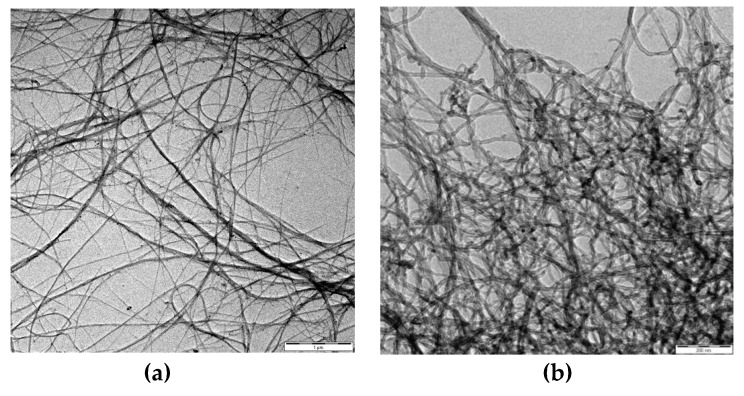
Transmission electron microscope images of: Rhenofit^®^ CNT-3 (**a**); and Rhenofit^®^ CNT-2 (**b**) from [60].

The SWCNTs and MWCNTs used in experiments were acquired from RheinChemie-Lanxess, Germany. They are water-based suspensions and can be directly used for production of formulations; their labels are Rhenofit^®^ CNT-3 and Rhenofit^®^ CNT-2, respectively. Table 2 shows the technical data of Rhenofit^®^ CNT-3 and Rhenofit^®^ CNT-2. Figure 1 shows their transmission electron microscope (TEM) images [60]. Agglomerates of CNTs can be observed in the pictures. 

#### 2.1.2. Cement and Sand

Ordinary Portland cement CEM Ⅰ 42.5 R (rapid hardening) manufactured by Irish Cement (Ireland) was used in the following experiments. Rapid hardening Portland cement (RHPC) is a special purpose cement used in concrete to achieve a higher rate of early strength development, compared to using normal cement. Its main particle size is 5–30 μm, it has a relative density of 2.75–3.20 g/cm^3^, and its apparent density is 0.9–1.5 g/cm^3^.

Standard silica sand from Glenview (Ireland) was used in the experiments. Sand was dried by placing into oven for 24 h at a temperature of 105–110 °C. The particle size of the sand was variable. To prepare cement mortars, the sand was sieved through a 600-μm sieve. This was carried out to reduce the voids formed when preparing specimens.

#### 2.1.3. Surfactant (SAA)

SAAs, such as TX10, SDS, GA, etc., are amphiphilic in nature and can be used as surface active agents. Structurally, they have a hydrophilic polar head and hydrophobic long tail [61]. They can be used to lower the surface tension between two liquids, between a gas and a liquid, or between a liquid and a solid. 

TX10 from Carl Roth GmbH + Co KG (Karlsruhe, Germany) was used in experiments. TX10 is known for having good dispersing performance on SWCNTs [38,49]. Furthermore, it was found by Carriçot et al. [62] that, for different surfactants, the optimum mass ratio of CNTs to dispersant are different. Therefore, the optimum dosage of TX10 to treat SWCNTs should be researched. Wang et al. [63] measured that the saturation adsorption ratio of surfactant vs. SWNT is ca. 0.004 mol/g. With the increase of the concentration of TX10, no SWCNTs in suspension liquid will react with extra TX10.

TX10 has an average molecular mass of 625, a density of 1.065 g/cm^3^, and a critical micelle concentration (CMC) of 0.22–0.24 × 10^−3^ moles/liter in water at 25 °C. One mole of TX10 is 625 g on average. The linear molecular formula is (C_2_H_4_O)nC_14_H_22_O. It is a nonionic surfactant. 

Therefore, 1 g of SWCNTs needs the addition of 0.004 mol TX10 (2.5 g) to reach saturation dispersion [63]. In this experimental set, the dosage of TX10 should be determined. The maximum concentration of SWCNTs is 0.06 wt% by the mass of cement. In each mix group, the amount of cement was 1833 g; thus, the amount of added SWCNTs could be calculated as around 1.0998 g. Thus, the dosage of TX10 was around 2.75 g. Considering TX10 may negatively affect the cementitious matrix, 2.75 g TX10 was used for all concentrations of SWCNTs (0.02 wt%, 0.04 wt%, and 0.06 wt%), and, for all concentration, SWCNTs were well dispersed. In general, the mass ratio of TX10 was 0.15 wt% by the mass of cement.

### 2.2. Specimens Preparation

#### 2.2.1. Mortar Mix Ratio

The Mix Ratio 1 proportion is shown in Table 3. Normal mortar specimens, consisting of cement, sand, and water, were prepared following Mix Ratio 1. However, for mortars treated by TX10, Mix Ratio 2 was adjusted, as shown in Table 4. This is discussed in Section 2.2.4.

Slump tests were conducted to determine the workability of cement mortar mixed according to the mix ratios. The slump test was carried out as prescribed by ASTM C 143-10 and BS 1881:103:1993. According to Jensen et al. [64], surfactant TX100 acts as a plasticizer to some extent. In the following tests, the slump test was carried out to ensure that the mortar mix has similar workability. Mortar was divided into two groups: untreated plain mortars and TX10-treated mortars. For plain mortars, the slump test result was 52 mm.

According to BS EN 206-1:2000, Table 5 gives the European classification of slump test. It can be concluded that the workability of mortar with Mix Ratio 1 belongs to S2 classification.

#### 2.2.2. Concentration of SWCNTs in Mortar Preparation

Ferro et al. [65] found that, when very small quantities of MWCNTs (i.e., 0.025–0.08% wt. cement) were added, the mechanical properties of cement composites materials were noticeably improved, and the quantity of CNTs generally matched with the mass of cement of mixing ratio. Therefore, in this study, three concentrations of SWCNT were used: 0.02 wt%, 0.04 wt%, and 0.06 wt% by the mass of cement. In addition, experiments studying the effect of MWCNTs on mechanical properties of CNTs were also carried out to see the difference of strength between speciments with SWCNTs and MWCNTs. The concentrations of MWCNTs were 0.00 wt%, 0.04 wt%, 0.08 wt%, and 0.12 wt% by the mass of cement.

#### 2.2.3. Mortar Mix Design without TX10 Treatment

Table 6 shows the experimental mix design of SWCNT-added mortars without treatment of TX10 and the quantities of the materials. The mixes were named according to: SWCNTXN, where SWCNT represents the material SWCNT; X stands for the concentration of SWCNTs with 0, 2, 4, 6 representing 0.00 wt%, 0.02 wt%, 0.04 wt%, 0.06 wt% respectively; and N represents no SAA added. 

Table 7 shows the experimental design of MWCNT-added mortars without treatment of TX10 and quantities of each materials.

Rhenofit^®^ CNT-3 and Rhenofit^®^ CNT-2 are water-based CNT suspensions; 99.4% and 96% of the solutions is water, respectively. Thus, during preparation of specimens, the water contained in the Rhenofit^®^ CNT-3 and Rhenofit^®^ CNT-2 should be subtracted. Table 6 and Table 7 show the mix proportion of the materials. Since Rhenofit^®^ CNT-3 and Rhenofit^®^ CNT-2 are water-based CNT suspensions, as the concentration of SWCNTs and MWCNTs increased, less water was needed. This was to keep the quantity of water in mortars uniform.

Mortars with no CNTs were set as control samples, to compare with CNT-containing mortars.

#### 2.2.4. Mortar Mix Design with TX10 Treatment

When TX10 was added as an additive in preparing mortars, it was found that TX10 works as a plasticizer; the workability was greatly improved when Mix Ratio 1 (1:3:0.7) was used. Therefore, the slump test was carried out to determine a new mix ratio for TX10 contained samples. The results show that, when the water/cement ratio was 0.55, the slump test result was 54 mm, similar to that of plain mortar. Therefore, for TX10-treated mortar, the new Mix Ratio 2 of cement:sand:water was 1:3:0.55.

Following Mix Ratio 2, mix designs for TX10 added cement mortars were set. Table 8 shows the experiment groups of mortar with TX10 treatment, and the quantity of each materials. The quantity of TX10 was 2.75 g; as mentioned in Section 2.1.3, the mass of TX10 is 0.15 wt% by the mass of cement. The mixes were named according to: SWCNTXS, where SWCNT represents for the material SWCNT; X stands for the concentration of SWCNTs with 0, 2, 4, and 6 representing 0.00 wt%, 0.02 wt%, 0.04 wt%, and 0.06 wt%, respectively; and S means SAA was mixed. As mentioned above, less water was needed as the concentration of SWCNTs increased.

Table 9 shows the experimental mix design of MWCNT-added mortars without treatment of TX10 and the quantities of the materials. It should be noted that, for all mix designs, the quantity of each material was overprepared for the required three cubes and three prisms. As TX10 was 0.15 wt% by the mass of cement, 2.75 g of TX10 was needed for MWCNT-added mortar.

#### 2.2.5. Mixing Process

For specimens without treatment of TX10, the mix process was as follows:

1. Certain quantities of water and Rhenofit^®^ CNT-3/CNT-2 suspension were mixed as Solution A, and were stirred.
2. All sand and half of Solution A were poured into the stir machine and stirred.
3. All cement was poured into the stir machine and stirred.
4. The rest of Solution A was poured into the stir machine.

For specimens with treatment of TX10, the mix process was as follows:

1. Certain quantities of water, Rhenofit^®^ CNT-3/CNT-2, and TX10 were mixed as Solution B, and were stirred.
2. All sand and half Solution B were poured into the stir machine and stirred.
3. All cement was poured into the stir machine.
4. The rest of Solution B was poured into the mixing machine.

Note that, when TX10 was added, foam was generated in the cement matrix, resulting in the increase of porosity of samples. The mass of specimens and bulk density decreased compared to the specimens with no TX10 added. This is discussed in Section 3.4.

After mixing of materials, the composites were filled into cube and prism molds. The size of the cube mold was 100 mm × 100 mm × 100 mm. The size of the prism mold was 40 mm × 40 mm × 160 mm. After 24 h, the mortar specimens were placed into curing container at a constant temperature of 23 ± 1 °C and relative humidity of 100% according to the ASTM C192. The curing times of SWCNT-added specimens were set as 3, 7, and 28 days. MWCNT-added cement-based specimens were set for 28 days.

### 2.3. Test Methods

#### 2.3.1. Compressive Test

To test the compressive strength of concrete, mortar cubes were prepared. After curing, the cubes were taken out of their curing containers, and the surfaces were mopped dry. A cube was placed at the center of the loading area of the compressive strength test machine. The loading rate was 0.6 MPa/s.

Loading stopped automatically when cubes were broken, and the compressive strength and force could be directly recorded. 

The compressive strength by knowing the maximum applied force was calculated as follows:

R_c_ = F_c_/10000 (1)

where R_c_ is the compressive strength in MPa; F_c_ is the maximum load at the time of fraction in N; and 10,000 is the area of the face of the cube (100 mm × 100 mm).

#### 2.3.2. Flexural Test

For flexural strength test, symmetrical three-point loading was used. In experiments, the 40 mm × 40 mm × 160 mm prisms were tested using three-point loading test. Prisms were placed in the loading area for processing the three-point bending test. Prisms were subjected to the load at a rate of 50 ± 10 N/s. The loading device was turned off when failure occurred. 

The flexural strength by knowing the maximum applied force was calculated as follows:

R_f_ = (1.5 × l × F_f_)/b^3^ (2)

where R_f_ is the flexural strength in MPa; b is the side of the square section of the prism in mm; F_f_ is the peak load applied to center of the prism at fracture in N; and l is the distance between supports in mm. For the three-point loading test device, l equals 100 mm and b equals 40 mm. During loading, the force applied was recorded. By using the maximum force recorded, the flexural strength can be calculated through Equation (2).

## 3. Results and Discussion

### 3.1. Comparison of Strength Properties of SWCNTs and MWCNTs Added Specimens

It is known that MWCNTs show better mechanical properties than SWCNTs, and many studies have been conducted to study the effect of MWCNTs on mechanical properties of cement composites. However, SWCNTs are more advantaged in enhancing electrical properties and thermal properties of cementitious materials, which give SWCNTs great potential for functional cement composites. This section compares the difference of strength properties of SWCNT- and MWCNT-added specimens when they were prepared by the same method. Table 10 shows the 28-day compressive strength and flexural strength of the two types of cement-based specimens with or without the treatment of TX10.

Table 10 shows that, for both specimens with no addition of CNTs and TX10, their strengths were around 30.5 MPa, resulting from the same mix ratio. With the addition of CNTs, the strength of both SWCNT–cement composites and MWCNT–cement composites decreased with the increase of concentration of CNTs when CNTs were directly used. This could be attributed to the increase of agglomerates of CNTs when the concentration of CNTs was increased. It can be observed that the compressive strength and flexural strength of MWCNT–cement composites were higher than those with SWCNTs. For instance, when the concentration of CNTs was 0.04 wt%, the compressive strength of MWCNTs was 27.46 MPa and the flexural strength was 6.96 MPa, 6.5% and 12.3% higher than those of SWCNT–cement composites. Thus, MWCNTs show better mechanical properties than SWCNTs, but the difference is relatively small. However, when CNTs were dispersed by TX10, SWCNT–cement composites show slightly better strength properties. When the concentration of CNTs was 0.04 wt%, the compressive and flexural strengths were 18.94 and 4.95 MPa, respectively, 2.6% and 3.2% higher than MWCNT–cement composites. This might be due to TX10, which shows the highest dispersing power when dispersing SWCNTs [49]. However, for MWCNTs, according to Luo et al. [39], TX10 was not suitable for dispersing MWCNTs. The reduction of strength after treatment of TX10 is discussed in following sections. Generally, MWCNTs show better mechanical properties, but the increase of strength properties is relatively small. There is potential to study the effect of SWCNTs on mechanical properties of cement-based composites. The following sections mainly discuss the strength properties of SWCNT-added cement composites.

### 3.2. Compressive Strength Test Results

To get compressive strength of mortars, three specimens were prepared. According to BS 1881-119:1983 and ASTM C 116-90, the average of the three values was taken as the representative compressive strength of the concrete. Table 11 shows the failure compressive strength of all specimens. The differences of SWCNTXN and SWCNTXS groups (where X equals 0, 2, 4, and 6) are water/cement ratio and TX10 content. Although the two groups had similar slump results, which indicates similar workability, the values of compressive strength shows great difference. Non-TX10-treated samples showed higher compressive strength than SAA treated samples. The cause might be the decrease of mass of cubes and prisms when TX10 was added. TX10 results in the formation of foam during the stir process, which retards or prevents the hydration of cement. The relationship between strength and mass of specimens are discussed in Section 3.3. In this section, the effects of SWCNTs and TX10 on sample strength are discussed.

#### 3.2.1. Specimens without Treatment of TX10

**Figure 2:**
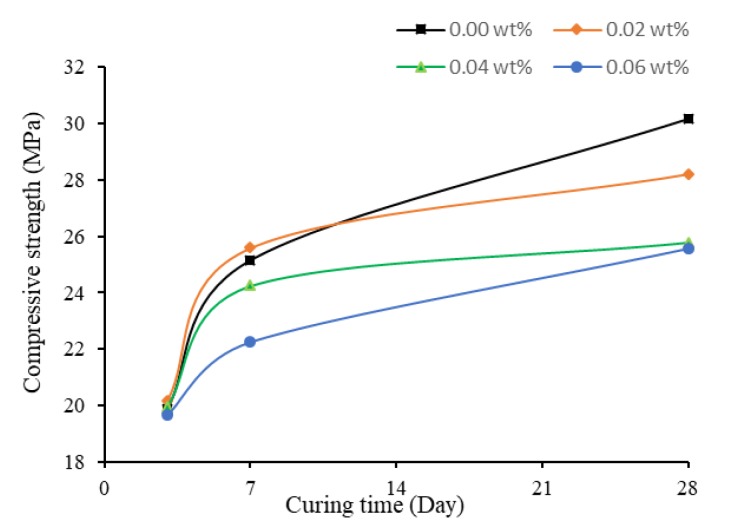
Compressive Strength of No TX10 contained mortar cubes.

The compressive strength of specimens with no TX10 added is plotted against time in Figure 2. The effect of SWCNTs on these specimens is discussed in this section.

Figure 2 shows variation of the compressive strength of specimens under four different SWCNT concentrations at different curing times. With no treatment of TX10, the higher was the concentration of the SWCNTs, the lower was the compressive strength of mortar cubes, which is similar to the results of Suprompituk et al. [66]. For plain cementitious mortar, the compressive strength could reach 30.15 Mpa after 28 days of curing, which decreased to 25.58 Mpa when the concentration of CNTs was increased to 0.06 wt%. The reason might be that, when SWCNTs were used directly, they easily tangled together, thus were not well dispersed in the cement matrix and formed agglomerates. This reduced the bond between the hydration products, and in turn the compressive strength of mortar was reduced. 

Moreover, at early age, the differences in compressive strength were small. At three days of curing, they shared similar compressive strength, all around 20 MPa. Thus, at early age, SWCNTs showed little effect on the mechanical properties of mortars. In addition, t the compressive strength of SWCNT0N and SWCNT2N after three and seven days of curing were similar, although the strength of SWCNT2N was slightly higher. However, at 28 days of curing, the strength of SWCNT2N was 6.43% lower than SWCNT0N. The reason the strength of 0.02 wt% SWCNT-containing specimens was higher at early stage might be the positive effect of SWCNTs on early hydration reaction of cement. This phenomenon that CNTs may affect the early hydration progress of cement, producing higher hydration rates, was also found by Markar and Beaudoin [67] and Markar et al. [68]. In general, the reduction of 28-day strength with the increase of concentration of SWCNTs was related to the agglomerates of CNTs.

**Figure 3:**
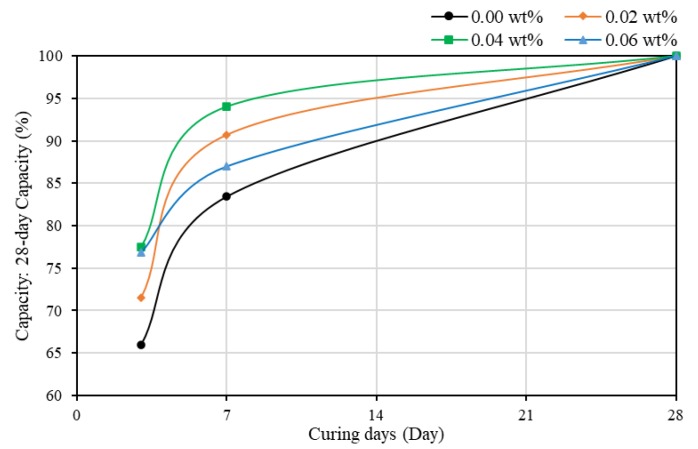
The evolution of the strength of mortars (no TX10) as a function of curing time.

To analyze the early hydration rate, the evolution of the strength of mortars is plotted in Figure 3. Adding CNTs improved the evolution of strength of mortar in gaining strength more quickly. At three days of curing, plain mortar gained around 66% strength. With the increase of the quantity of SWCNTs, the hydration rate was improved. Obvious differences occurred at seven days of curing; the compressive strength could reach around 90–94% of 28-day strength, which was higher than the 83.4% for SWCNT0N. However, when the concentration of CNTs reached 0.06 wt%, the percentage of gained strength at seven days of curing was around 87%, between plain mortars and SWCNT2N. This might be the negative effect of agglomerates. In general, adding SWCNTs can make cement composites gain strength more quickly at an early age. In addition, it was also found that, when the concentration of SWCNTs was 0.04 wt%, the evolution of compressive strength of samples was the highest.

**Figure 4:**
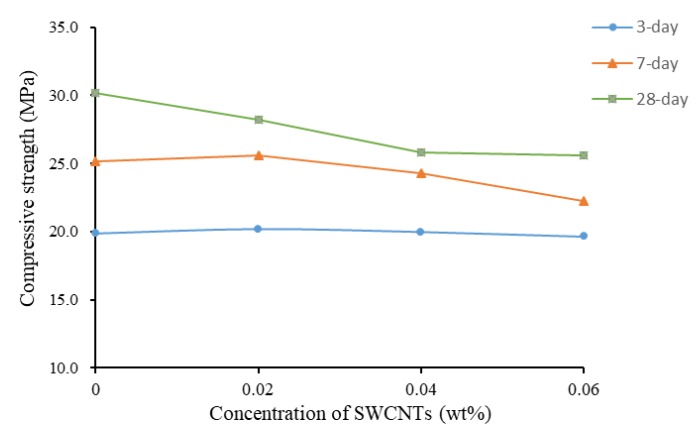
Compressive strength as a function of concentration of SWCNTs (No TX10).

Figure 4 shows the effect of concentration of SWCNTs on compressive strength at different curing times. It clearly shows that the higher was the concentration, the lower was the compressive strength. After 28 days of curing, specimens showed a higher compressive strength than seven- or three-day cured specimens. At three days of curing, the compressive strengths of specimens at the four concentrations showed similar values, around 20 MPa.

#### 3.2.2. Specimens with Treatment of TX10

When TX10 was added and mixed, the variation pattern of strength changed. The most obvious change shown in Table 11 is that the compressive strength of SWCNTXS (where X equals 0, 2, 4, and 6) was much lower than that of SWCNTXN (where X equals 0, 2, 4, and 6) at each curing time. The reasons might be the change of water/cement ratio and the effect of TX10 on the specimens. For samples with the addition of TX10, their masses were less than those of non-TX10-treated specimens because, when TX10 was added, much foam was generated in the cement paste, as mentioned in Section 3.2, causing high porosity in mortars, and in turn the decrease of the specimens’ bulk density. Moreover, TX10 might have partly decreased the bond of cement reaction. Based on this situation, the effect of SWCNTs and variation pattern on strength properties of mortars under the dispersion method of TX10 were studied. 

**Figure 5:**
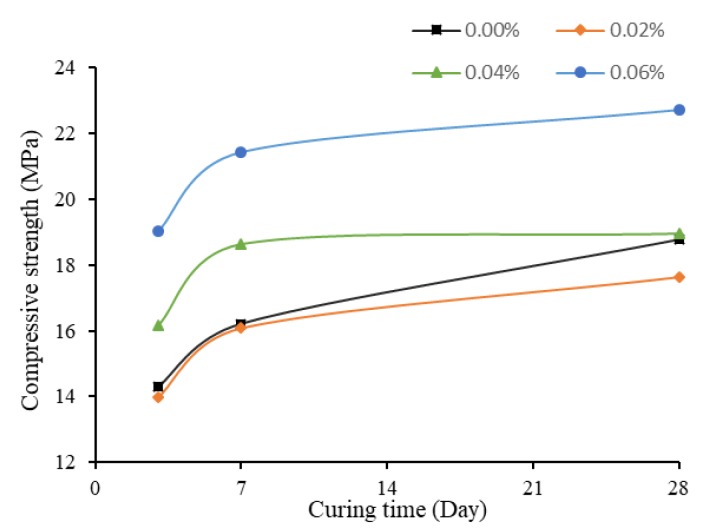
Compressive Strength of TX10 added mortar cubes.

**Figure 6:**
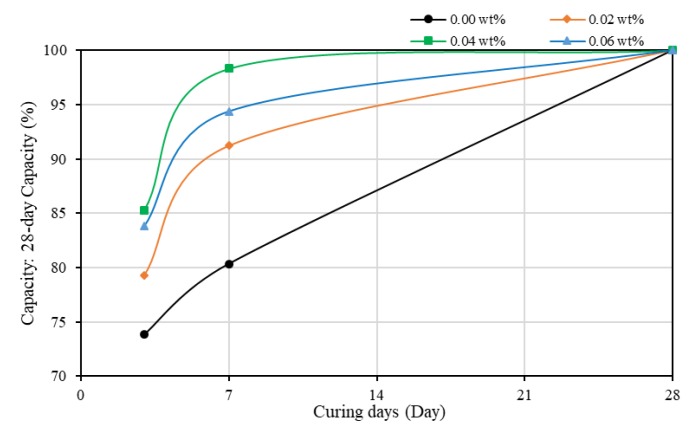
The evolution of the strength of mortars (SAA) as a function of curing time.

Figure 5 shows that the compressive strength increased with the increase of concentration of SWCNTs; the higher was the concentration, the higher was the compressive strength. After 28 days of curing, the compressive strength of SWCNT6S increased 21% compared to SWCNT0S. The reason might be that TX10 changed the surface energy of SWCNT suspensions; getting SWCNTs well dispersed in water allowed the unique properties of CNTs to show up. Moreover, it should be noted that, at three days of curing, different from SWCNTXN (where X equals 0, 2, 4, and 6), which had no TX10 added and shared similar compressive strength of 20 MPa, the strength of TX10-treated mortars increased with the increase of concentration of CNTs, although the strength of SWCNT2S was slightly lower than that of SWCNT0S. Furthermore, Figure 6 shows that, with the addition of SWCNTs, the rapid hardening phenomenon of mortar was more obvious, even though CEM |, a type of rapid hardening cement, was used in experiments. At seven days of curing, when the concentration of SWCNTs was 0.04 wt%, the compressive strength could reach over 95% of the 28-day compressive strength. Compared to Figure 3, when the concentration of SWCNTs was 0.06 wt%, the percentage of strength gained could reach approximately 94%, which was higher than the 87% of non-TX10-treated specimens. Moreover, Figure 3 and Figure 6 show that, for plain mortars with no CNTs added, TX10 decreased the hydration rate to some extent. The strength gained percentage decreased from 83.4% to 80% when TX10 was added.

**Figure 7:**
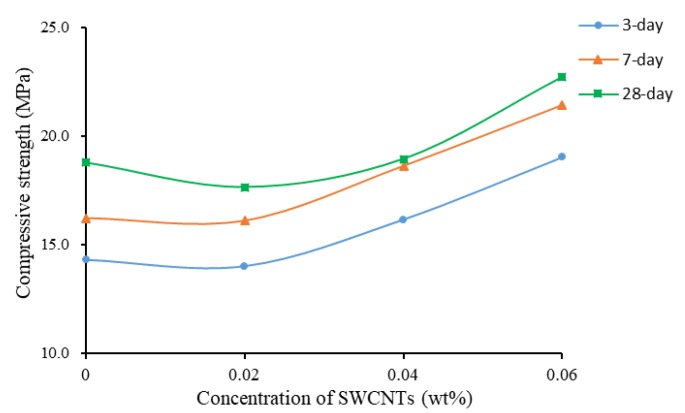
Compressive strength as a function of concentration of SWCNTs.

Figure 7 shows the compressive strength as a function of concentration of SWCNTs. The compressive strength was reduced initially, but then, with the increase of the concentration of SWCNTs, the compressive strength increased, reaching the peak when the concentration was 0.06 wt%. The reduction of strength might be due to the change of fluidity when CNTs were added, which requires more research. Thus, TX10 as a SAA worked well on dispersing SWCNTs in cement matrix, and SWCNTs helped enhance the interface bond force among aggregates and cement matrix. The agglomerates that might obstruct the cement pastes were reduced. Therefore, SAA is a useful dispersion method regardless of the negative effect on mechanical properties of cement composites. However, it should be noted that the addition of TX10 produced foam in the cement, resulting in the high porosity of mortars, causing a decrease in the bulk density of samples, and in turn the overall decrease of strength. Therefore, in future works, defoamer should be considered to study the possibility of improving the reduction of strength when TX10, or another SAA is added.

### 3.3. Flexural Strength Test Results

To obtain the flexural strength of concrete, three specimens were prepared. According to BS 1881-119:1983 and ASTM C 116-90, the average of the three values was taken as the representative flexural strength. The results of tested specimens are shown in Table 12.

#### 3.3.1. Specimens without Treatment of TX10

**Figure 8:**
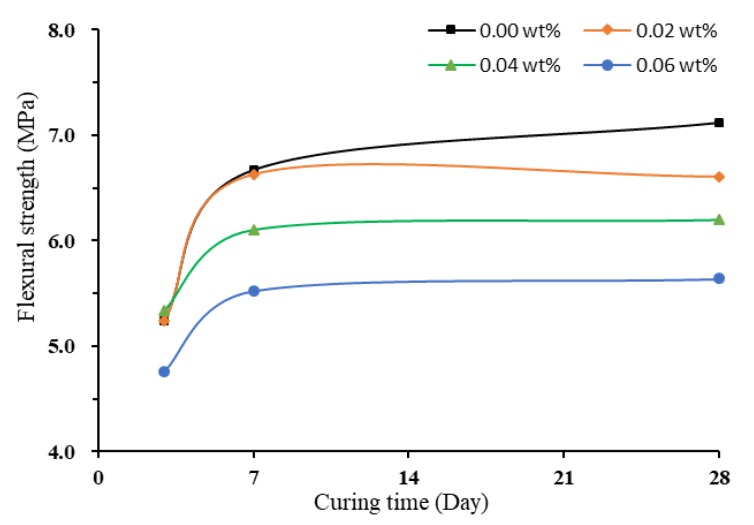
Flexural strength of non-TX10-added mortar prisms.

Similar to the compressive strength of non-TX10-treated specimens, the flexural strength decreased with the increase of the concentration of SWWCNTs. Figure 8 shows the increase of flexural Strength of non-TX10-containing mortar prisms as a function of curing time. SWCNT-containing mortars could reach high flexural strength at early stage (seven days). After seven days of curing, the flexural strength reached stable value and continued curing gained little increase in flexural strength. The reason is that, with the addition of SWCNTs, the rapid hardening phenomenon of mortar was more obvious, even though early hardening cement was used. At 28 days of curing, SWCNT0S showed 6.75% increase in flexural strength compared to the seven-day strength, but, for SWCNTXS (where X equals 2, 4, and 6), the increase was little, only from 0.91% to 2.17%. This might be the crack-bridging effect in cement/CNTs composites produced by CNTs. CNTs have the ability to prevent the development of cracks [65,69]. TEM is needed for further works to analyze the crack-bridging effect of CNTs and to study the effect of CNTs on hydration products of cement/CNTs composites.

**Figure 9:**
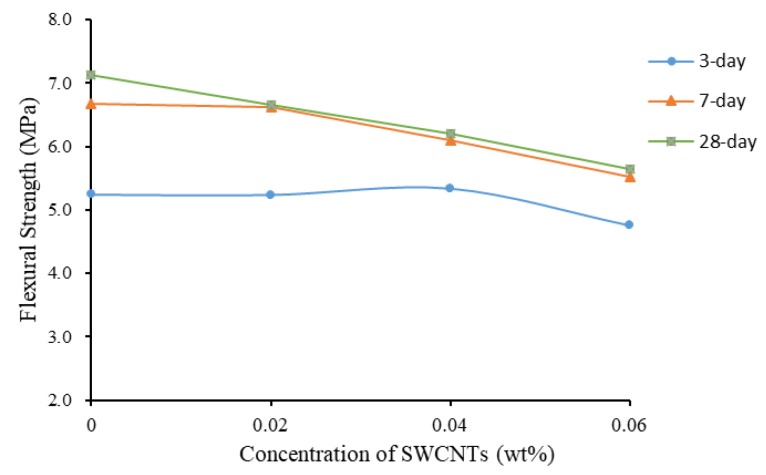
Flexural strength as a function of concentration of SWCNTs (No TX10).

In addition, Figure 9 shows that the flexural strength decreased with the increase of the concentration of SWCNTs. It can also be observed that, when SWCNTs were added, the values of flexural strength after 7 and 28 days of curing were close for all concentrations of SWCNTs used.

#### 3.3.2. Specimens with Treatment of TX10

**Figure 10:**
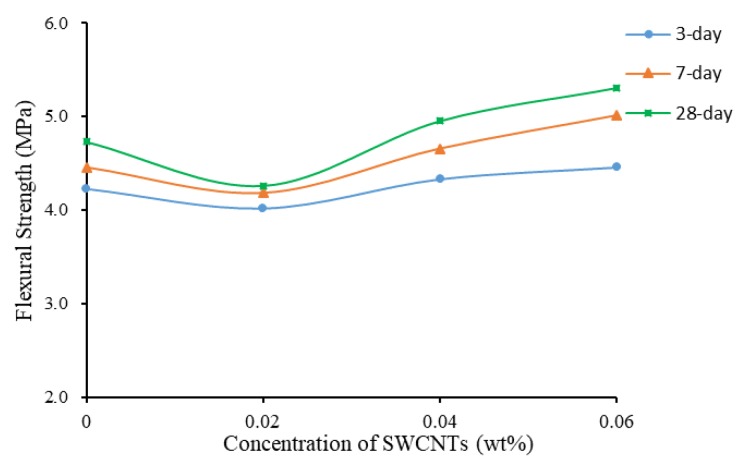
Flexural strength as a function of concentration of SWCNTs (TX10).

When TX10 was added and mixed, the variation pattern of flexural strength changed. Figure 10 shows that the flexural strength was high initially, adding SWCNTs caused a slight reduction, and then the flexural strength increased with the increase of the concentration of SWCNTs. After 28 days of curing, flexural strength of SWCNT6S reached 5.31 MPa, 19.77% and 10.92% higher compared to flexural strength of SWCNT2S and SWCNT0S at 28 days of curing, respectively. This variation pattern was different to that when TX10 was not added, as shown in Figure 9 and Figure 10. The dispersed CNTs showed positive effect on flexural strength of mortars.

**Figure 11:**
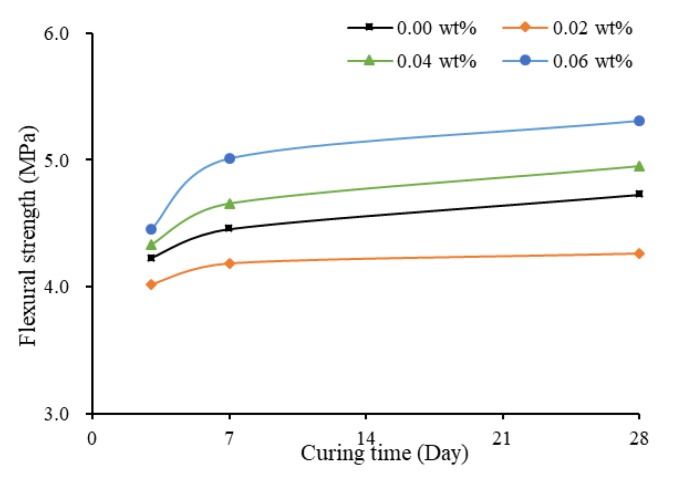
Flexural strength of TX10-added mortar prisms.

Figure 11 shows that the flexural strength increased with the increase of concentration of SWCNTs, although the value of flexural strength of SWCNT0S was between those of SWCNT2S and SWCNT4S. By comparing Figure 8 and Figure 11, it can be observed that, with the addition of TX10, the flexural strength of mortar with 0.06 wt% CNTs was the highest, resulting from the positive effect of CNTs. This finding was opposite to that of non-TX10-added mortars, where plain mortar presented the highest level.

### 3.4. Relationship between Strength and Bulk Density of Cement Based Specimens 

In the experiments, the mass of each tested cube and prism was recorded. According to the size of mortars and prisms, the bulk density of each specimens could be calculated. Table 13 shows the average bulk density of each set of mortars.

**Figure 12:**
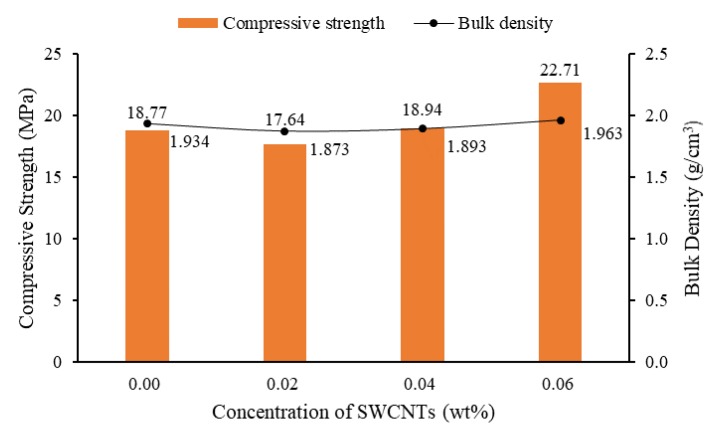
Variation of 28-day compressive strength and bulk density of TX10-treated specimens as a function concentration of SWCNTs.

Table 13 shows that for non-TX10-added mortars, the bulk density was around 2.102–2.141 g/cm^3^, higher than those of TX10-treated mortars (1.884–1.998 g/cm^3^). This result proves that the porosity of mortars was increased due to the addition of TX10, which caused the reduction of strength of mortars. In addition, the strength of mortars was related to the bulk density of mortars. For instance, Figure 12 shows the variation of 28-day compressive strength and bulk density of TX10-treated specimens as a function of the concentration of SWCNTs. The average bulk density of plain cube mortars (no SWCNTs added) was 1.934 g/cm^3^, which was reduced to 1.873 g/cm^3^ with the increase of the concentration of CNTs to 0.02 wt%, and then increased to 1.893 and 1.963 g/cm^3^ when the concentration of CNTs was increased to 0.04 wt% and 0.06 wt%, respectively. The variation pattern of 28-day compressive strength was similar to the variation of bulk density of specimens, i.e., the higher was the bulk density, the higher was the compressive strength.

Therefore, the strength (MPa)/mass of specimens (g) ratio was considered to standardize the law. It was found that the compressive and flexural strengths were related to the mass of the mortar cube/prism. Table 14 shows some of the data: average compressive and flexural strengths after seven days of curing are listed as illustration.

Table 14 shows that both the compressive strength and the flexural strength was related to the mass of the specimens. When SWCNTs were directly used, the general trend of strength/mass ratio was downward, which was same trend as for strength. When TX10 was added to pretreat SWCNTs, the ratio saw a slight increase when the concentration of SWCNTs increased, also similar to the variation pattern of strength.

The value of comp/mass of cubes ratio was close to the flex/mass ratio of prisms. The differences between the two ratios were generally less than 5%. Therefore, this relationship can be used to estimate the compressive strength (flexural strength) when the mass of the specimens and flexural strength (compressive strength) are known. However, in some cases, the difference reached 8–15%, which could be observed from results of SWCNTXS (where X equals 0, 2, 4, or 6) at three days of curing (Table 15). Its cause might be the errors in the process of preparing the specimens

Generally, it can be concluded that, when SWCNTs and MWCNTs were directly used to prepare mortars, specimens’ strength decreased with the increase of concentration of CNTs due to the increase of CNTs’ agglomerates. It was found that MWCNT-added specimens showed better strength properties than SWCNT-added specimens due to MWCNTs having better mechanical properties, although the differences of the strength of the cement composites were small. The effect of SWCNTs on strength properties of cement composites was mainly studied. When TX10, as a surfactant, was applied, the variation pattern was changed. The compressive and flexural strengths generally increased with the increase of concentration of SWCNTs, although TX10 produced a negative effect on cement composites by increasing porosity, causing a decrease in the bulk densities of mortar specimens. It was found that SWCNTs could help cement composites gain high strength more quickly at the early stage. In addition, it was found that the strength was related to the mass of the specimens. 

## 4. Conclusions

This paper presents the effect of SWCNTs (Rhenofit^®^ CNT-3) on the strength properties of cement composites. In addition, the effect of the addition of TX10 (SAA) on the strength properties of cement composites is also discussed. The main results of this investigation are summarized as follows:

1. Directly using Rhenofit^®^ CNT-3/CNT-2 (without addition of TX10) in cement matrix reduced the compressive strength of cement composites, resulting from the agglomeration of CNTs. The compressive and flexural strengths decreased as a function of the concentration of CNTs. Plain mortars showed the highest strength at this stage.

2. When TX10 was not added, MWCNTs (Rhenofit^®^ CNT-2) added to cement-based specimens showed better strength properties than SWCNTs, resulting from the higher mechanical properties of MWCNTs. When TX10 was applied, the strength properties of SWCNT–cement composites were higher than those of MWCNT–cement composites, because TX10 was not suitable in dispersing MWCNTs.

3. When TX10 was applied, the compressive and flexural strengths of cement composites were greatly reduced compared to the case where no TX10 was used. In addition, the bulk density and mass of specimens were also reduced, from 2.102–2.141 to 1.884–1.998 g/cm^3^. The reduction of strength and bulk density could be attributed to the formation of foam during the specimen preparation process and the retardation of hydration caused by TX10. 

4. Even though strength reduction of cement composites occurred when TX10 was used to disperse SWCNTs, when the effect of SWCNTs on strength properties of cement composites was studied at this stage, it was found that the general variation trend of compressive and flexural strengths was upward; the strength increased with the increase of the concentration of SWCNTs. Conversely to when no TX10 was added to specimens, the maximum compressive and flexural strengths occurred when the concentration of SWCNTs was 0.06 wt%. 

5. SWCNTs affected the early hydration progress of cement. SWCNTs could produce high hydration rates at early age: at seven days of curing, the compressive and flexural strengths could reach over 90% of the 28-day strength.

6. The variation of compressive and flexural strengths was related to the mass (bulk density) of mortar specimens. They shared similar variation patterns: the strength of specimens increased or decreased as a function of the mass of specimens.

7. The flexural strength/mass of cubes ratio was close to compressive strength/mass of prisms ratio, with difference less than 10%. This relation could be used to estimate the strength of specimens.

For future work, TEM should be applied to study the failure modes of specimens and the effect mechanism of CNTs on hydration products of cement/CNTs composites.

## Figures and Tables

**Table 1:** Differences between SWCNTs and MWCNTs.

	SWCNT	MWCNT
**Structure**	Diameter: 0.5–3.0 nm	Diameter: 5–100 nm
	Length: 100 nm–1 cm	Length: 100 nm–1 cm
**Typical Young’s modulus**	1.3 TPa	1.8 TPa
**Tensile strength**	Up to 53 GPa	Up to 63 GPa
**Electrical conductivity**	10,000 S/cm	6000 S/cm
**Heat conductivity**	Max. 6000 W/m·k	Max. 3000 W/m·k

**Table 2:** Brief technical data sheet of Rhenofit^®^ CNT-3/Rhenofit^®^ CNT-2.

	Rhenofit^®^ CNT-3	Rhenofit^®^ CNT-2
**Composition**	0.2 wt% single wall carbon nanotubes	2 wt% Multi wall carbon nanotubes
	0.4 wt% dispersant agent in water	4 wt% dispersant agent in water
**Appearance**	stable black suspension	stable black suspension
**Odor**	no odor	no odor
**Compatibility**	can be mixed with liquid systems and some polar organic solvents	can be mixed with liquid systems and some polar organic solvents
**pH Value**	7.7	8–10

**Table 3:** Mix Ratio 1 used for mortars (no TX10).

1:3:0.7 Mix Ratio of Mass		
Cement	Sand	Water
1	3	0.70

**Table 4:** Mix Ratio 2 used for mortars (TX10 added).

1:3:0.55 Mix Ratio of Mass		
Cement	Sand	Water
1	3	0.55

**Table 5:** Classification of Workability and Magnitude of Slump.

Classification of Workability	Slump (mm)
S1	10–40
S2	50–90
S3	100–150
S4	≥160

**Table 6:** Mix design of SWCNT-added mortar without treatment of TX10.

Mix Name	Cement (g)	Sand (g)	Water (g)	SWCNT (%wt, cement)	Rhenofit^®^ CNT-3 (g)	Total Mass of Solution (g)
SWCNT0N	1833	5499	1283.1	0.00	0.0	1283.1
SWCNT2N	1833	5499	1099.8	0.02	183.3	1283.1
SWCNT4N	1833	5499	916.4	0.04	366.7	1283.1
SWCNT6N	1833	5499	733.2	0.06	549.9	1283.1

**Table 7:** Mix design of mortar MWCNTs added without treatment of TX10.

Mix Name	Cement (g)	Sand (g)	Water (g)	MWCNT (%wt, cement)	Rhenofit^®^ CNT-2 (g)	Total Mass of Solution (g)
MWCNT0N	1842	5529	1289.4	0.00	0.0	1289.4
MWCNT4N	1842	5529	1106.1	0.04	36.8	1289.4
MWCNT8N	1842	5529	922.8	0.08	73.6	1289.4
MWCNT12N	1842	5529	739.5	0.12	110.4	1289.4

**Table 8:** Mix design of SWCNT-added mortars with treatment of TX10.

Mix Name	Cement (g)	Sand (g)	Water (g)	SWCNT (%wt, cement)	Rhenofit^®^ CNT-3 (g)	TX10 (g)
SWCNT0S	1833	5499	1008.2	0.00	0.0	2.75
SWCNT2S	1833	5499	824.85	0.02	183.3	2.75
SWCNT4S	1833	5499	641.55	0.04	366.6	2.75
SWCNT6S	1833	5499	458.25	0.06	549.9	2.75

**Table 9:** Mix design of MWCNT-added mortar with treatment of TX10.

Mix Name	Cement (g)	Sand (g)	Water (g)	SWCNT (%wt, cement)	Rhenofit^®^ CNT-3 (g)	TX10 (g)
MWCNT0S	1842	5529	1013.1	0.00	0.0	2.76
MWCNT2S	1842	5529	976.3	0.04	36.8	2.76
MWCNT4S	1842	5529	939.5	0.08	73.6	2.76
MWCNT6S	1842	5529	902.7	0.12	110.4	2.76

**Table 10:** Strength properties of SWCNTs and MWCNT-added specimens after 28 days of curing.

SWCNTs					MWCNTs				
SWCNTs Concentration	No TX10		TX10 Added		MWCNTs Concentration	No TX10		TX10 Added	
	Com	Fle	Com	Fle		Com	Fle	Com	Fle
**0.00 wt%**	30.15	7.12	18.77	4.73	**0.00 wt%**	30.57	7.08	18.12	4.59
**0.02 wt%**	28.21	6.66	17.64	4.26	**0.04 wt%**	29.26	6.96	18.46	4.79
**0.04 wt%**	25.79	6.20	18.94	4.95	**0.08 wt%**	27.46	6.94	21.71	4.91
**0.06 wt%**	25.58	5.64	22.71	5.31	**0.12 wt%**	24.86	6.62	21.65	5.06

**Table 11:** Compressive strength test results of SWCNT-added specimens.

Compressive Strength (MPa)												
Mix Name	3 Days			Mean	7 Days			Mean	28 Days			Mean
**SWCNT0N**	18.70	19.24	21.71	19.88	25.01	25.16	25.28	25.15	29.45	29.73	31.26	30.15
**SWCNT2N**	19.03	20.46	21.06	20.18	25.34	25.55	25.89	25.59	28.07	28.09	28.48	28.21
**SWCNT4N**	18.73	20.47	20.75	19.98	23.13	24.74	24.89	24.25	24.94	25.68	26.76	25.79
**SWCNT6N**	17.84	18.92	22.22	19.66	21.51	22.54	22.70	22.25	25.42	25.65	25.67	25.58
**SWCNT0S**	13.98	14.44	14.46	14.29	15.84	16.24	16.55	16.21	18.56	18.86	18.89	18.77
**SWCNT2S**	13.66	13.88	14.45	13.99	15.76	15.85	16.67	16.09	17.55	17.64	17.72	17.64
**SWCNT4S**	15.97	16.12	16.37	16.15	18.43	18.71	18.72	18.62	18.64	18.96	19.22	18.94
**SWCNT6S**	18.88	18.98	19.23	19.03	20.76	21.09	22.43	21.43	21.87	22.94	23.32	22.71

**Table 12:** Flexural strength test results of SWCNT-added specimens.

Flexural Strength (MPa)												
Mix Name	3 Days			Mean	7 Days			Mean	28 Days			Mean
**SWCNT0N**	5.02	5.34	5.35	5.24	6.47	6.70	6.84	6.67	6.76	7.11	7.52	7.12
**SWCNT2N**	5.12	5.25	5.35	5.24	6.46	6.71	6.73	6.63	6.08	6.60	7.15	6.66
**SWCNT4N**	5.13	5.41	5.46	5.34	5.94	6.12	6.27	6.11	6.12	6.24	6.25	6.20
**SWCNT6N**	4.55	4.63	5.10	4.76	5.19	5.57	5.79	5.52	5.29	5.48	6.14	5.64
**SWCNT0S**	4.11	4.25	4.32	4.23	4.31	4.40	4.66	4.46	4.69	4.71	4.78	4.73
**SWCNT2S**	4.00	4.02	4.03	4.02	3.99	4.25	4.31	4.18	4.20	4.27	4.32	4.26
**SWCNT4S**	4.27	4.32	4.40	4.33	4.54	4.64	4.79	4.66	4.68	4.97	5.21	4.95
**SWCNT6S**	4.16	4.58	4.63	4.46	4.66	4.88	5.50	5.01	5.21	5.29	5.42	5.31

**Table 13:** Average bulk density of mortar specimens.

Types	Curing Days	0.00 wt%		0.02 wt%		0.04 wt%		0.06 wt%	
		Cubes	Prisms	Cubes	Prisms	Cubes	Prisms	Cubes	Prisms
**Non-TX10-treated**	**3**	2.094	2.164	2.122	2.164	2.115	2.082	2.107	2.129
	**7**	2.106	2.164	2.114	2.082	2.109	2.152	2.115	2.156
	**28**	2.105	2.074	2.114	2.176	2.108	2.121	2.105	2.078
	**Mean**	2.102	2.134	2.117	2.141	2.111	2.118	2.109	2.121
**TX10-treated**	**3**	1.882	1.891	1.902	1.871	1.939	1.867	1.999	2.000
	**7**	1.887	1.875	1.878	1.902	1.943	1.918	2.024	2.031
	**28**	1.934	1.871	1.873	1.855	1.893	1.852	1.963	1.965
	**Mean**	1.901	1.879	1.884	1.876	1.925	1.879	1.995	1.998

**Table 14:** Compressive (flexural) strength/mass ratio of cubes (prisms) at seven days of curing.

Mix Name	Cube Mass (g)	Comp (MPa)	Prism Mass (g)	Flex (MPa)	Comp/ Mass	Flex/ Mass	Difference
SWCNT0N	2106	25.15	554	6.67	0.01194	0.01205	0.921%
SWCNT2N	2114	25.59	533	6.63	0.01211	0.01244	2.725%
SWCNT4N	2109	24.25	551	6.11	0.01149	0.01108	3.568%
SWCNT6N	2115	22.25	552	5.52	0.01052	0.01000	4.943%
SWCNT0S	1887	16.21	480	4.46	0.00859	0.00928	8.032%
SWCNT2S	1878	16.09	487	4.18	0.00857	0.00859	0.233%
SWCNT4S	1943	18.62	491	4.66	0.00949	0.00949	0.000%
SWCNT6S	2024	21.43	498	5.01	0.01059	0.01006	5.005%

Comp represents compressive strength, and Flex represents flexural strength.

**Table 15:** Compressive (flexural) strength/mass ratio of cubes (prisms) at three days of curing.

Mix Name	Cube Mass (g)	Comp (MPa)	Prism Mass (g)	Flex (MPa)	Comp/ Mass	Flex/ Mass	Difference
SWCNT0S	1882	14.29	484	4.23	0.00759	0.00874	15.152%
SWCNT2S	1902	14.00	479	4.02	0.00736	0.00839	13.995%
SWCNT4S	1939	16.15	478	4.33	0.00833	0.00906	8.764%
SWCNT6S	1999	19.03	512	4.46	0.00952	0.00871	8.508%

Where the comp represents compressive strength, and the flex represents flexural strength.
